# Hypocitraturia and Risk of Bone Disease in Patients With Kidney Stone Disease

**DOI:** 10.1002/jbm4.10786

**Published:** 2023-06-27

**Authors:** Calyani Ganesan, I‐Chun Thomas, Maria E Montez‐Rath, Glenn M Chertow, John T Leppert, Alan C Pao

**Affiliations:** ^1^ Department of Medicine, Division of Nephrology Stanford University Palo Alto CA USA; ^2^ Division of Nephrology and Department of Urology Veterans Affairs Palo Alto Health Care System Palo Alto CA USA; ^3^ Department of Urology Stanford University Palo Alto CA USA

**Keywords:** FRACTURE PREVENTION AND GENERAL POPULATION STUDIES, FRACTURE RISK ASSESSMENT, OSTEOPOROSIS, SCREENING

## Abstract

Patients with kidney stone disease are at higher risk for bone disease. Hypocitraturia is common in patients with kidney stone disease and a key risk factor for stone recurrence. In this retrospective cohort study, we sought to determine whether hypocitraturia is also a risk factor for incident bone disease in patients with kidney stone disease. We used nationwide data from the Veterans Health Administration and identified 9025 patients with kidney stone disease who had a 24‐hour urine citrate measurement between 2007 and 2015. We examined clinical characteristics of patients by level of 24‐hour urine citrate excretion (<200, 200–400, and >400 mg/d) and the time to osteoporosis or fracture according to 24‐hour urine citrate excretion level. Almost one in five veterans with kidney stone disease and a 24‐hour urine citrate measurement had severe hypocitraturia, defined as <200 mg/d. Patients with severe hypocitraturia were at risk for osteoporosis or fracture (hazard ratio [HR] = 1.23; confidence interval [CI] 1.03–1.48), but after adjustment for demographic factors, comorbid conditions, and laboratory abnormalities associated with hypocitraturia, the association was no longer statistically significant (HR = 1.18; CI 0.98–1.43). Our results in a predominantly male cohort suggest a modest association between hypocitraturia and osteoporosis or fracture; there are likely to be other explanations for the potent association between kidney stone disease and diminished bone health. © 2023 The Authors. *JBMR Plus* published by Wiley Periodicals LLC on behalf of American Society for Bone and Mineral Research.

## Graphical abstract

There is a modest association between hypocitraturia and osteoporosis or fracture in kidney stone patients; there are likely other explanations for the potent association between kidney stones and bone disease.

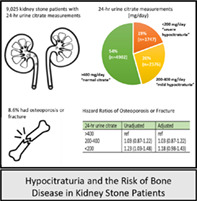

## Introduction

Almost one in four veterans with kidney stone disease experience a fracture or diagnosis of osteoporosis.^[^ ^1^, ^2^ ^]^ However, currently no screening guidelines exist for identifying poor bone health in this population. In a recent cross‐sectional analysis, we found an association between low 24‐hour urine citrate excretion and prevalent bone disease in veterans with kidney stone disease.^[^ ^2^ ^]^ Low urine citrate excretion may be an early indicator of a net acid load to the body and may signal an attempt to conserve or retain alkali (citrate) by the kidney.^[^ ^3^ ^]^ To further maintain acid–base balance, alkali from bone may be mobilized to compensate for an acid load, ultimately leading to bone loss over time.^[^ ^4^ ^]^ If the presence of hypocitraturia can identify which patients with kidney stone disease are at risk for developing bone disease, this information could alert clinicians to screen such patients for osteopenia or osteoporosis.^[^ ^5^, ^6^, ^7^, ^8^ ^]^ Furthermore, diagnosis of hypocitraturia could prompt clinicians to prescribe alkali supplementation to increase urine citrate excretion not only for reducing stone recurrence^[^ ^5^, ^6^, ^7^, ^8^ ^]^ but also for improving bone health.^[^ ^4^ ^]^ It remains unknown whether the presence of hypocitraturia can be used to identify patients with kidney stone disease who are at risk for developing osteoporosis or fracture.

We used nationwide data from the Veterans Health Administration (VHA) and identified 9025 patients with kidney stone disease who had a 24‐hour urine citrate measurement between 2007 and 2015. Our objective was to examine demographics, comorbid illness, and laboratory characteristics of patients with varying levels of urine citrate excretion and to test whether patients with lower levels of urine citrate excretion were at risk for an incident diagnosis of osteoporosis or fracture. We hypothesized that patients with hypocitraturia are more likely to be at risk for osteoporosis or fracture.

## Materials and Methods

### Data and study population

The Stanford University School of Medicine Institutional Review Board and the Veterans Affairs Research and Development Committee approved this study and waived the requirements for informed consent because the data were de‐identified and presented in aggregate. We accessed national VHA data stored in the Corporate Data Warehouse, which is hosted by the Veterans Affairs Informatics and Computing Infrastructure, to identify patients with kidney stone disease who received care in one of 130 VHA facilities across the United States from January 1, 2007, to December 1, 2015. We defined patients with kidney stone disease as those with one or more inpatient encounters that included International Classification of Diseases, Ninth Revision and Tenth Revision (ICD‐9 and ICD‐10) codes for kidney or ureteral stones, two or more outpatient encounters for kidney or ureteral stones, or one or more kidney or ureteral stone procedures within 1 year using Current Procedural Terminology (CPT) codes.^[^ ^2^, ^9^, ^10^ ^]^ Each patient was counted once, at the time of his or her first qualification for kidney stone disease during the observation period. Veterans with incident and recurrent kidney stone disease were included. Next, we selected patients with kidney stone disease who completed a 24‐hour urine citrate measurement within 6 months of their index stone diagnosis. If a patient had more than one 24‐hour urine citrate measurement within the observation period, we selected the measurement closest to the index stone diagnosis.

### Covariates and outcomes

We abstracted patient demographics (age, sex, and race/ethnicity), relevant comorbid conditions (diabetes, enteric disease, metastatic cancer, hypogonadism, osteoporosis, and history of fracture), and laboratory data (serum creatinine, bicarbonate, and potassium concentrations). All serum and urine laboratory measurements were identified using Logical Observation Names and Codes within the time period of 6 months before and 1 week after completion of a 24‐hour urine citrate measurement to ensure that serum and urine labs correlated with 24‐hour urine citrate measurement. If a patient had more than one laboratory measurement within the observation period, we selected the closest laboratory measurement to the 24‐hour urine citrate measurement. The main outcome was time to osteoporosis or fracture. We treated 24‐hour urine citrate excretion as a categorical variable (<200 mg/d and 200–400 mg/d and >400 mg/d).

### Statistical analysis

To evaluate the risk of osteoporosis or fracture according to level of 24‐hour urine citrate excretion, we categorized patients by level of 24‐hour urine citrate and performed a Cox proportional hazards regression analysis for incident diagnosis of osteoporosis or fracture after stone diagnosis. We adjusted for demographics and body size (age, sex, and Quételet body mass index), comorbid conditions (type 2 diabetes mellitus, metastatic cancer, hypogonadism, and enteric disease defined as Crohn's disease, ulcerative colitis, and celiac disease) and serum measurements (serum creatinine, bicarbonate, and potassium concentrations). We performed a subgroup analysis that included men with an accompanying 24‐hour urine creatinine excretion of 15–25 mg per kg of body weight per day and women with an accompanying 24‐hour urine creatinine excretion of 10–20 mg per kg of body weight per day.^[^ ^11^ ^]^ We conducted all statistical analyses with SAS, version 9.4 (SAS Institute Inc., Cary, NC, USA).

## Results

### Clinical characteristics of patients with low 24‐hour urine citrate excretion

**Fig. 1:**
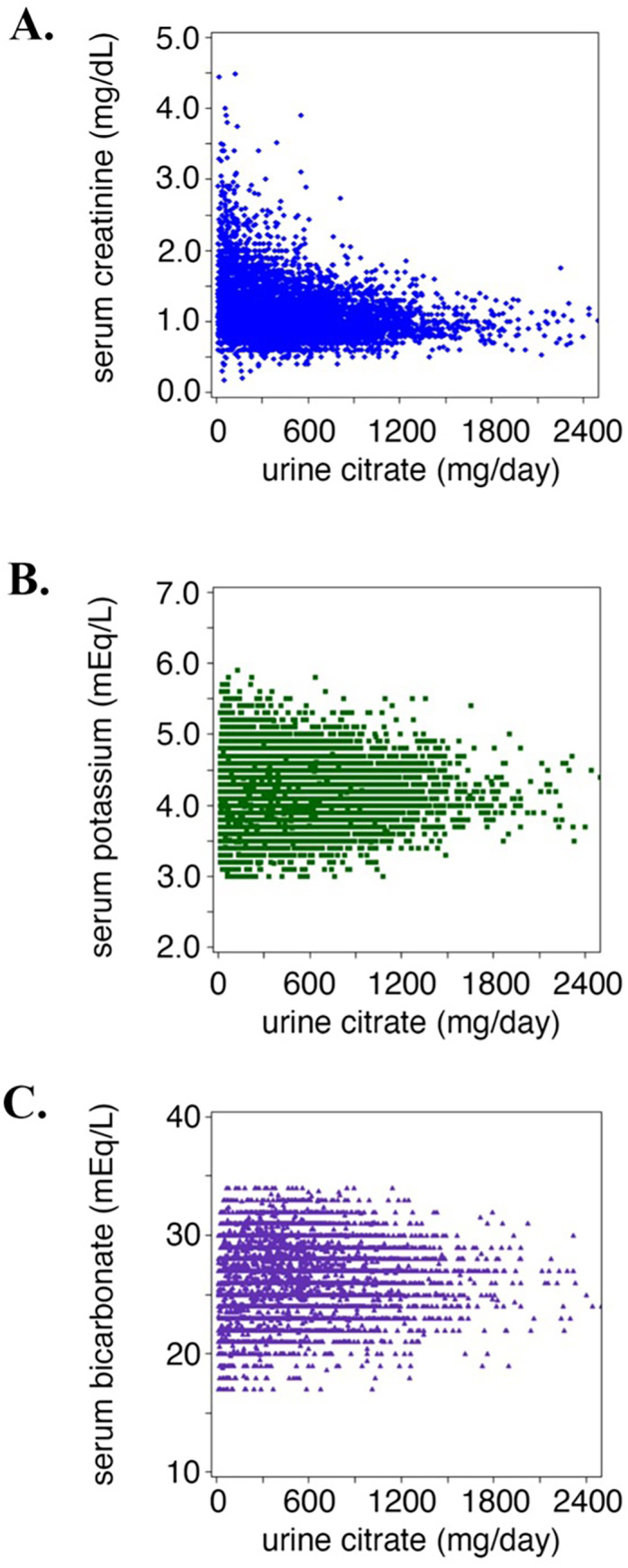
Baseline serum laboratory measurements of patients with kidney stone disease.

We identified 9025 unique patients with kidney stone disease who completed a 24‐hour urine citrate measurement from January 1, 2007, to December 31, 2015. In this cohort, 19.4% (1747/9025) had a urine citrate <200 mg/d, 26.3% (2376/9025) had a urine citrate between 200 and 400 mg/d, and 54.3% (4902/9025) had a urine citrate >400 mg/d (Table 1). Patients with a urine citrate <200 mg/d had a mean (SD) age of 59.9 (13.3) years; the majority were men (91.6%) and were white (78.8%). Patients with a normal 24‐hour urine citrate measurement, defined as >400 mg/d, had a mean (SD) age of 57.2 (12.3) years; the majority were men (92.8%) and were white (82.5%) (Table 1). For patients with a urine citrate <200 mg/d, the proportion of patients with a diagnosis of type 2 diabetes mellitus was 33.7% (589/1747) compared with 25% (595/2376) of those with a urine citrate between 200 and 400 mg/d, and 30.4% (1492/4902) of those with a urine citrate >400 mg/d. The proportion of patients with a diagnosis of enteric disease was 2.1% (37/1747) with a urine citrate <200 mg/d compared with 1.2% (29/2376) of those with a urine between 200 and 400 mg/d, and 0.7% (36/4902) of those with a urine > 400 mg/d. Finally, Figure 1 shows the distribution of baseline serum creatinine, serum potassium, and serum bicarbonate concentration by 24‐hour urine citrate level for patients in our cohort.

**Table 1:** Baseline Characteristics of Patients With Kidney Stone Disease by 24‐Hour Urine Citrate Level

	24‐hour urine citrate measurement total patients, *n* (%)		
Characteristics	<200 mg/d (*n* = 1747)	200–400 mg/d (*n* = 2376)	>400 mg/d (*n* = 4902)
Age (years), mean (SD)	59.9 (13.3)	56.5 (13.6)	57.2 (12.3)
Sex			
Male	1601 (91.6)	2196 (92.4)	4550 (92.8)
Female	146 (8.4)	180 (7.6)	352 (7.2)
Race/ethnicity			
White	1376 (78.8)	1926 (81.1)	4034 (82.3)
Black	164 (9.4)	165 (6.9)	360 (7.3)
Other	190 (10.9)	253 (10.6)	449 (9.2)
Body mass index			
<19	21 (1.2)	15 (0.6)	15 (0.3)
19–25	335 (19.2)	361 (15.2)	564 (11.5)
26–30	542 (31.0)	807 (34.0)	1529 (31.2)
>30	742 (42.5)	1014 (42.7)	2434 (49.7)
Comorbid conditions			
Metastatic cancer	28 (1.6)	14 (0.6)	29 (0.6)
Type 2 diabetes mellitus	589 (33.7)	595 (25.0)	1492 (30.4)
Enteric disease^a^	37 (2.1)	29 (1.2)	36 (0.7)
Hypogonadism	53 (3.0)	81 (3.4)	164 (3.3)
Charlson comorbidity index, mean (SD)	2.5 (2.4)	1.6 (1.9)	1.4 (1.7)
Laboratory values			
24‐hour urine citrate (mg/d), mean (SD)	110.9 (53.4)	300.9 (57.2)	730.4 (310.3)
Serum creatinine (mg/dL), mean (SD)	1.3 (0.6)	1.1 (0.3)	1.0 (0.3)
Serum potassium (mEq/L), mean (SD)	4.2 (0.5)	4.1 (0.4)	4.2 (0.4)
Serum bicarbonate (mEq/L), mean (SD)	25.7 (3.2)	26.7 (2.8**)**	26.8 (2.7)

Abbreviation: *n* = number of patients; SD = standard deviation.

^a^ Crohn's disease, ulcerative colitis, and celiac disease.

### Association between 24‐hour urine citrate excretion and osteoporosis or fracture

We examined the incidence of osteoporosis or fracture after a stone diagnosis in patients by level of 24‐hour urine citrate excretion. We excluded patients with a prior history of osteoporosis, hip fracture, and non‐hip fracture to ensure that we could capture incident diagnoses of bone disease. Approximately 8.7% (756/8674) of patients developed osteoporosis or fracture, with a mean time to event (SD) of 2.2 (1.3) years after stone diagnosis. Patients with urine citrate <200 mg/d had higher risk of osteoporosis or fracture (hazard ratio [HR] = 1.23; confidence interval [CI] 1.03–1.48) compared with patients with urine citrate measurement >400 mg/d by univariate analysis (Table 2). After adjustment for demographic factors, comorbid conditions, and laboratory abnormalities associated with hypocitraturia, the association was no longer statistically significant (HR = 1.18; CI 0.98–1.43). After adjusting for level of 24‐hour urine calcium, the association between hypocitraturia and osteoporosis or fracture did not materially change. We did not adjust for anti‐osteoporosis medications (ie, bisphosphonates, parathyroid hormone analogs, and receptor activator of NF‐κB [RANK] ligand inhibitors) given the very low prevalence of these medications in our cohort (*n* = 14, less than 1%).

**Table 2:** Multivariable‐Adjusted Hazard Ratios for Incident Diagnosis of Osteoporosis or Fracture

Variable	Model A HR (95% CI)	Model B HR (95% CI)	Model C HR (95% CI)	Model D HR (95% CI)
24‐hour urine citrate (mg/d)				
>400	ref	ref	ref	ref
200–400	1.03 (0.87–1.22)	1.02 (0.86–1.22)	1.03 (0.87–1.23)	1.03 (0.87–1.22)
<200	1.23 (1.03–1.48)	1.19 (0.99–1.43)	1.21 (1.00–1.46)	1.18 (0.98–1.43)
Age, per 10 years		1.08 (1.02–1.14)	1.08 (1.02–1.14)	1.07 (1.01–1.13)
Male		0.57 (0.45–0.73)	0.59 (0.46–0.76)	0.59 (0.45–0.76)
Race				
White		ref	ref	ref
Black		0.64 (0.47–0.88)	0.65 (0.48–0.89)	0.65 (0.48–0.89)
Other		0.78 (0.60–1.01)	0.78 (0.60–1.01)	0.78 (0.6–1.01)
Body mass index				
19–25		ref	ref	ref
<19		1.47 (0.73–2.95)	1.45 (0.72–2.90)	1.45 (0.72–2.91)
26–30		0.78 (0.63–0.97)	0.78 (0.63–0.96)	0.78 (0.63–0.95)
>30		0.79 (0.64–0.96)	0.79 (0.65–0.97)	0.77 (0.63–0.95)
Comorbid condition				
Metastatic cancer			1.32 (0.96–1.81)	1.31 (0.96–1.80)
Type 2 diabetes mellitus			1.07 (0.90–1.26)	1.07 (0.91–1.27)
Enteric disease			1.43 (0.79–2.59)	1.43 (0.79–2.60)
Hypogonadism			1.05 (0.69–1.59)	1.06 (0.70–1.60)
Laboratory values				
1/serum creatinine, per 0.1 mg/dL				1.01 (0.99–1.04)
Serum bicarbonate, per 1 mEq/L				0.99 (0.97–1.02)
Serum potassium, per 1 mEq/L				0.94 (0.79–1.13)

Abbreviation: HR = hazard ratio; CI = confidence interval.

Model A: univariate analysis of 24‐hour urine citrate measurement.

Model B: adjusted for demographic factors.

Model C: adjusted for demographic factors and relevant comorbid condition.

Model D: adjusted for demographic factors, relevant comorbid conditions, and serum measurements.

We also performed a subgroup analysis in which we included only patients with a 24‐hour urine citrate measurement from an adequate 24‐hour urine collection, defined as an accompanying 24‐hour urine creatinine excretion of 15–25 mg/kg of body weight per day for men and 10–20 mg/kg of body weight per day for women. For this analysis, the following patients were excluded: 20.6% (1131/5464) of patients with a 24‐hour urine citrate <200 mg/d; 26.6% (1456/5464) of patients with a 24‐hour urine citrate 200–400 mg/d; and 52.6% (2877/5464) of patients with a 24‐hour urine citrate >400 mg/d. Most were excluded because of an undercollected urine sample: 94.5% (1069/1131) of patients with a 24‐hour urine citrate <200 mg/d; 93.3% (1359/1456) of patients with a 24‐hour urine citrate 200–400 mg/d; and 87.8% (2528/2877) of patients with a 24‐hour urine citrate >400 mg/d. In the remaining cohort (*n* = 3300), patients with urine citrate <200 mg/d had higher risk of osteoporosis or fracture (HR = 1.40; CI 1.06–1.89) compared with patients with urine citrate measurement >400 mg/d by univariate analysis. After adjustment for demographic factors, comorbid conditions, laboratory abnormalities associated with hypocitraturia, and level of 24‐hour urine calcium excretion, the association remained statistically significant (HR = 1.40; CI 1.02–1.93, Supplemental Table S1).

## Discussion

In this large national cohort of veterans with kidney stone disease, we found that a higher fraction of veterans with severe hypocitraturia were diagnosed with osteoporosis or fracture within 5 years of a stone diagnosis. However, hypocitraturia alone was not a strong risk factor for an incident diagnosis of osteoporosis or fracture after adjustment for demographic factors and comorbid conditions. More veterans with severe hypocitraturia (urine citrate <200 mg/d) were older, female, lean, and had a diagnosis of metastatic cancer or enteric disease compared with those with higher levels of urine citrate excretion. We surmise that hypocitraturia may better predict poor bone health when these clinical characteristics are present in these patients. Future prospective studies will ultimately be needed to test whether urine citrate can be used to stratify persons with kidney stone disease for risk of osteoporosis or fracture.

The diagnosis of hypocitraturia is consequential for several reasons. First, for patients with kidney stone disease, it will identify those who will benefit from alkali supplementation to lower the risk of recurrence of calcium stones. Second, hypocitraturia may be an early sign of acid retention in patients with chronic kidney disease or gastrointestinal alkali loss.^[^ ^1^, ^3^, ^12^ ^]^ In such patients, alkali from bone is mobilized for acid buffering, which will compensate for overt metabolic acidosis but will also place them at risk for lower bone strength.^[^ ^13^ ^]^ Indeed, prior studies have demonstrated a link between lower serum bicarbonate concentration and lower bone mineral density as well as a correlation between higher bone mineral density and alkali therapy.^[^ ^4^, ^14^, ^15^ ^]^ Hypocitraturia may indicate a state of positive acid balance, but we suggest that other indicators such as a low urine pH, in conjunction with low urine citrate, may be needed to capture a signal for early acid retention or diminished bone health.^[^ ^3^ ^]^

This study has several strengths. To our knowledge, this is the first study to test whether the presence of hypocitraturia can be used to identify patients with kidney stone disease who are at risk for osteoporosis or fracture. This study included clinical and laboratory characteristics in a large cohort of veterans with kidney stone disease and hypocitraturia. We had access to national VHA data, which includes demographics, inpatient and outpatient diagnostic claims, and laboratory results from patients in the largest integrated national health care system in the US. This cohort was diverse in age, race/ethnicity, geographic location, and presence of comorbid conditions. We also tried to account for adequacy of 24‐hour urine collection for patients who had a 24‐hour urine citrate measurement by including a subgroup analysis in which only men with 24‐hour urine creatinine excretion of 15–25 mg/kg per day and women with 24‐hour urine creatinine excretion of 10–20 mg/kg per day were included. In this analysis, the association between hypocitraturia and incident bone disease was stronger, perhaps because we eliminated patients who excreted less urine citrate from an inadequate collection rather than from actual hypocitraturia.

This study has several limitations. First, we conducted this study in a cohort from the VHA, which is comprised mostly of men. Our study was not adequately powered to address sex differences in acid–base handling or urinary citrate excretion in patients with kidney stone diesase.^[^ ^16^ ^]^ Second, we used only diagnosis codes rather than radiologic imaging to determine the presence of kidney stones or fractures. Therefore, we may have missed kidney stones or fractures that may be clinically silent or nearly so, eg, small lower‐pole kidney stones or vertebral compression fractures. Third, we characterized patients with kidney stone disease who completed a 24‐hour urine citrate measurement, which is not a commonly ordered test, and inclusion of such patients may introduce selection bias to our study.^[^ ^17^ ^]^ Nonetheless, there is no way to characterize hypocitraturia except by 24‐hour urine measurement, and this study represents the largest cohort available to characterize this patient population. Fourth, we relied on a 24‐hour urine citrate measurement to categorize patients with kidney stone disease. It is possible that a 24‐hour urine citrate measurement in our cohort is not precise because of under‐ or overcollection of a 24‐hour urine sample. We tried to account for adequacy of 24‐hour urine collection for patients who had a 24‐hour urine citrate measurement in a subgroup analysis, by selecting patients who had an adequate creatinine excretion rate, calculated as 24‐hour urine creatinine per kilogram of patient body weight. Finally, we did not account for medical treatment of kidney stone disease that might concomitantly lower risk of bone disease in our cohort, such as administration of alkali or thiazide diuretics. However, we demonstrated in a prior study that most veterans with kidney stone disease are not prescribed alkali therapy after a low 24‐hour urine citrate measurement, suggesting that the modest association between hypocitraturia and bone disease in our cohort would not change even if we had excluded patients who received alkali therapy.^[^ ^9^ ^]^

In conclusion, our results in a predominantly male cohort suggest a modest association between hypocitraturia and osteoporosis or fracture. However, there are likely to be other explanations for the association between kidney stone disease and diminished bone health. Future studies are needed to test whether urine citrate in conjunction with other known risk factors for bone disease could be used to identify patients with kidney stone disease who could benefit from bone health screening.

## Author Contributions

**Calyani Ganesan:** Conceptualization; investigation; methodology; writing – original draft; writing – review and editing. **I‐Chun Thomas:** Data curation; formal analysis; project administration; software. **Maria E Montez‐Rath:** Data curation; formal analysis; project administration; supervision. **Glenn M Chertow:** Conceptualization; formal analysis; investigation; methodology; resources; writing – review and editing. **John T Leppert:** Conceptualization; formal analysis; funding acquisition; investigation; methodology; project administration; resources; supervision; writing – original draft; writing – review and editing. **Alan C Pao:** Conceptualization; formal analysis; funding acquisition; investigation; methodology; project administration; resources; supervision; writing – original draft; writing – review and editing.

## Conflicts of Interest

The authors have nothing to disclose.

### Peer Review

The peer review history for this article is available at https://www.webofscience.com/api/gateway/wos/peer-review/10.1002/jbm4.10786.

## Supporting information


**Table S1.** Multivariable‐adjusted hazard ratios for incident diagnosis of osteoporosis or fracture in men with a creatinine excretion rate of 15–25 mg/kg per day and women with a creatinine excretion rate of 10–20 mg/kg per day.

## Data Availability

The data that support the findings of this study are available from the Veterans Health Administration Corporate Data Warehouse, which is hosted by the Veterans Affairs Informatics and Computing Infrastructure. Restrictions apply to the availability of these data and only be accessed with the permission of the Veterans Health Administration.
