# Supplementary material for: Hypocitraturia and Risk of Bone Disease in Patients With Kidney Stone Disease
Source: JBMR Plus. 2023 Jun 27;7(9):e10786. doi: 10.1002/jbm4.10786 (PMC10494504; doi:10.1002/jbm4.10786)
Supplement: Supplementary file 1 — Table S1. Multivariable‐adjusted hazard ratios for incident diagnosis of osteoporosis or fracture in men with a creatinine excretion rate of 15–25 mg/kg per day and women with a creatinine excretion rate of 10–20 mg/kg per day. [file JBM4-7-e10786-s001.docx]

**Supplemental Table 1.** **Multivariable adjusted hazard ratios for incident diagnosis of osteoporosis or fracture in men with a creatinine excretion rate of 15-25 mg/kg per day and women with a creatinine excretion rate of 10-20mg/kg per day**

| **Variable** | **Model A**  **HR (95% CI)** | **Model B**  **HR (95% CI)** | **Model C**  **HR (95% CI)** | **Model D**  **HR (95% CI)** |
| --- | --- | --- | --- | --- |
| 24-hour urine citrate, mg/day  > 400  200-400  < 200 | ref  1.12 (0.86-1.45)  1.41 (1.06-1.89) | ref  1.11 (0.85-1.44)  1.39 (1.04-1.86) | ref  1.06 (0.81-1.39)  1.35 (0.99-1.83) | ref  1.09 (0.83-1.43)  1.40 (1.02-1.93) |
| Age, per 10 years |  | 1.08 (0.99-1.19) | 1.08 (0.98-1.19) | 1.09 (0.99-1.20) |
| Male |  | 0.47 (0.34-0.66) | 0.49 (0.34-0.70) | 0.48 (0.33-0.69) |
| Race  White  Black  Other |  | ref  0.81 (0.51-1.28)  0.98 (0.68-1.41) | ref  0.86 (0.54-1.36)  0.99 (0.68-1.42) | ref  0.88 (0.55-1.41)  1.00 (0.69-1.44) |
| Body Mass Index  19-25  <19  26-30  >30  Comorbid Condition  Metastatic cancer  Type 2 Diabetes mellitus  Enteric Disease  Hypogonadism  Laboratory Values  1/serum creatinine, per 0.1 mg/dL  Serum bicarbonate, per 1 mEq/L  Serum potassium, per 1 mEq/L  24-hour urine calcium, per 50mg |  |  | ref  1.13 (0.35-3.68)  0.87 (0.63-1.19)  0.66 (0.48-0.91)  1.65 (1.00-2.71)  0.93 (0.71-1.21)  0.79 (0.25-2.48)  1.38 (0.75-2.54)  1.02 (0.97-1.06)  1.00 (0.96-1.04)  0.91 (0.68-1.20) | ref  1.14 (0.35-3.71)  0.86 (0.62-1.18)  0.64 (0.47-0.89)  1.64 (1.00-2.70)  0.93 (0.71-1.21)  0.80 (0.26-2.52)  1.38 (0.75-2.54)  1.01 (0.97-1.06)  1.00 (0.96-1.04)  0.91 (0.69-1.21)  1.03 (0.97-1.09) |

Model A: univariate analysis of 24-hour urine citrate measurement

Model B: adjusted for demographic factors

Model C: adjusted for demographic factors, relevant comorbid condition, and serum measurements

Model D: adjusted for demographic factors, relevant comorbid conditions, serum measurements and 24-hour urine calcium measurement
